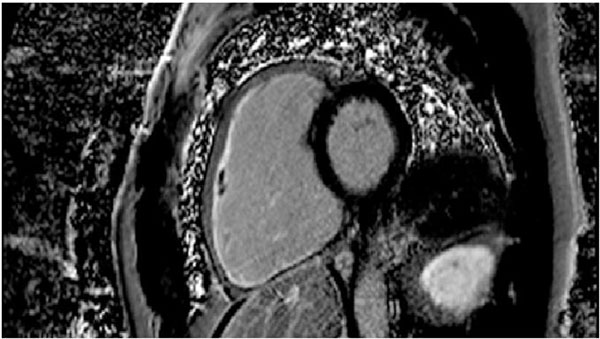# Low diagnostic yield of Late Gadolinium Enhancement (LGE) in screening patients with suspected Arrhythmogenic Right Ventricular Cardiomyopathy (ARVC) by Cardiovascular Magnetic Resonance (CMR)

**DOI:** 10.1186/1532-429X-14-S1-P141

**Published:** 2012-02-01

**Authors:** Frans van Hoorn, Janice Paproski, Danna Spears, Elsie T Nguyen, Rachel M Wald, Sebastian Ley, Felipe S Torres, Narinder S Paul, Bernd J Wintersperger, Andrew M Crean

**Affiliations:** 1 Medical Imaging, Toronto General Hospital, Toronto, ON, Canada; 2 Cardiology, Toronto General Hospital, Toronto, ON, Canada; 3 Medical Imaging, Sunnybrook Hospital, Toronto, ON, Canada

## Background

ARVC is a rare potentially life-threatening inherited disease of the desmosome. Right ventricular wall motion abnormalities combined with right ventricular dilatation or impaired function on CMR are diagnostic criteria for this disease. The role of LGE in this disease is still under debate. This study was performed to investigate the yield of LGE by CMR in patients referred with suspected ARVC.

## Methods

Retrospective review of CMR data sets of patients referred for assessment of possible ARVC between 01/2005 and 12/2009 meeting following inclusion criteria: a) clinical assessment by a staff electrophysiologist; b) stack of LGE images in at least one plane. Clinical charts were reviewed and 2010 revised Task Force Criteria (TFC) used to score the clinical probability of ARVC. Presence or absence of right ventricular wall motion abnormalities (RVWMA), right and left ventricular end-diastolic volume indexed for body surface area (RVEDVi, LVEDVi), ejection fraction (RVEF, LVEF) and presence of right ventricular and left ventricular LGE (RVLGE, LVLGE) were recorded.

## Results

156 patients met inclusion criteria. Mean patient age was 43 ± 14 yrs, 71 were males. Mean (± SD) RVEDVi, RVEF, LVEDVi, and LVEF were 85 ± 20 ml/m2, 54 ± 7%, 79 ± 16 ml/m2 and 58 ± 8% respectively.

ARVC diagnosis, based on TFC, was definite in 3 patients, borderline in 13 patients and possible in 26 patients. Twelve patients (8%) in total demonstrated LGE. Of these twelve, 5 patients (3%) had LVLGE and 5 patients (3%) RVLGE. Two patients (1.3%) had biventricular LGE.

Patients with presence of RVLGE had marginally lower RVEF but similar right ventricular volumes compared to patients without RVLGE (47±8 vs 54±6, p=0.05 and 92±22 vs 84±20, p=0.39 for RVEF and RVEDVi respectively). RVWMA, seen in 38 patients (24%) was significantly related to RVLGE (6/38 vs 1/118, p=0.02).

Causes of isolated RVLGE were RV infarction post right coronary angioplasty in 1 patient and post ablation fibrosis in 2 patients. Only 1 patient with isolated RVLGE was borderline positive for ARVC by TFC. Two patients with isolated LVLGE had borderline ARVC and 1 had possible ARVC. The 2 patients showing biventricular LGE had definite and borderline ARVC respectively. Only 6 patients (4%) in our cohort had both LGE and a final diagnosis of definite, borderline or possible ARVD by TFC.

## Conclusions

In our cohort of patients with suspected ARVC there was a low incidence of LGE. Administration of gadolinium should be directed towards patients showing either RV wall motion abnormalities and impaired RV systolic function, or cases classified as definite / borderline / possible ARVC by modified Task Force criteria.

## Funding

None

**Table 1:** Relationship between modified Task Force Criteria for ARVD and presence of LGE

(by 2010 Task Force Criteria)	1. Definite ARVC (n=3)	2. Borderline ARVC (n=13)	3. Possible ARVC (n=26)	4. Not 1, 2 or 3 (n=112)
RV LGE +ve	0	1	0	4
LV LGE +ve	0	2	1	2
BiV LGE +ve	1	1	0	0

**Figure 1:**